# Mycb and Mych stimulate Müller glial cell reprogramming and proliferation in the uninjured and injured zebrafish retina

**DOI:** 10.1242/dev.203062

**Published:** 2024-07-26

**Authors:** Mi-Sun Lee, Jonathan Jui, Aresh Sahu, Daniel Goldman

**Affiliations:** Michigan Neuroscience Institute and Department of Biological Chemistry, University of Michigan, Ann Arbor, MI 48109, USA

**Keywords:** Stem cell, Regeneration, Myc, Apoptosis, Protein synthesis, Cell cycle, Zebrafish

## Abstract

In the injured zebrafish retina, Müller glial cells (MG) reprogram to adopt retinal stem cell properties and regenerate damaged neurons. The strongest zebrafish reprogramming factors might be good candidates for stimulating a similar regenerative response by mammalian MG. Myc proteins are potent reprogramming factors that can stimulate cellular plasticity in differentiated cells; however, their role in MG reprogramming and retina regeneration remains poorly explored. Here, we report that retinal injury stimulates *mycb* and *mych* expression and that, although both Mycb and Mych stimulate MG reprogramming and proliferation, only Mych enhances retinal neuron apoptosis. RNA-sequencing analysis of wild-type, *mych^mut^* and *mycb^mut^* fish revealed that Mycb and Mych regulate ∼40% and ∼16%, respectively, of the genes contributing to the regeneration-associated transcriptome of MG. Of these genes, those that are induced are biased towards regulation of ribosome biogenesis, protein synthesis, DNA synthesis, and cell division, which are the top cellular processes affected by retinal injury, suggesting that Mycb and Mych are potent MG reprogramming factors. Consistent with this, forced expression of either of these proteins is sufficient to stimulate MG proliferation in the uninjured retina.

## Teaser

**Summary:** Analysis of the effects of retinal injury in wild-type, mutant and transgenic zebrafish reveal that Mycb and Mych are potent reprogramming factors in Müller glia.

## INTRODUCTION

Neurodegenerative diseases of the human retina, such as glaucoma and macular degeneration, are accompanied by neuron loss and blindness. In contrast, neuron death in the injured zebrafish retina triggers a regenerative response that replenishes lost neurons (Goldman, 2014; Wan and Goldman, 2016). Key to retina regeneration in zebrafish are Müller glial cells (MG), a radial glial cell type that normally participates in maintenance of retinal homeostasis and structure (Reichenbach and Bringmann, 2013; MacDonald et al., 2015). Zebrafish MG are the only retinal cell type to respond to neuron death by undergoing a reprogramming event that stimulates asymmetric cell division and production of a multipotent progenitor for retinal neuron regeneration (Fausett and Goldman, 2006; Ramachandran et al., 2010b; Nagashima et al., 2013; Powell et al., 2013). Remarkably, this regenerative response is sufficient to restore sight to blinded fish (Hammer et al., 2021). In contrast, MG in the mammalian retina exhibit a gliotic response to retinal injury that is characterized by cell swelling and increased expression of intermediate filament proteins (Bringmann et al., 2009).

A number of recent studies indicate that forced expression of certain transcription factors and signaling molecules in mouse MG can stimulate their transdifferentiation into specific retinal neuron types; however, these reprogrammed MG rarely divide and do not have characteristics of a retinal stem cell (Yao et al., 2018; Todd et al., 2021, 2022; Boudreau-Pinsonneault et al., 2023; Le et al., 2024). Furthermore, the regenerated neurons are often immature and retain some glial characteristics. Thus, there is a need to identify additional factors that can impart stem cell character to mammalian MG and allow for robust neuron regeneration without depleting the MG pool. We think these factors can be most readily identified in a pro-regenerative system, such as the zebrafish retina, in which MG reprogramming and production of multipotent MG-derived progenitors is robust. Furthermore, we suspect that the most potent reprogramming factors will be sufficient to stimulate MG reprogramming and proliferation in the uninjured retina. Thus, we aim to identify potent reprogramming factors underlying retina regeneration in zebrafish with the hope that they will help stimulate a regenerative response in a diseased or injured human retina.

MG RNA sequencing (RNAseq) and retinal single-cell (sc)RNAseq have revealed most of the genes that are differentially expressed in MG as they reprogram and prepare for cell division in the injured retina (Sifuentes et al., 2016; Hoang et al., 2020; Lee et al., 2020; Sahu et al., 2021). These differentially regulated genes are referred to as MG's regeneration-associated transcriptome. Functional analysis of some of these regeneration-associated genes revealed their role in regulating MG proliferation and neuron regeneration (Fausett et al., 2008; Nelson et al., 2012, 2013; Wan et al., 2012; Conner et al., 2014; Gorsuch et al., 2017; Wan and Goldman, 2017; Elsaeidi et al., 2018; Iribarne et al., 2019; Lee et al., 2020; Sharma et al., 2020; Campbell et al., 2021; Sahu et al., 2021; Mitra et al., 2022). However, except for the Notch signaling pathway, the impact these genes have on the regeneration-associated transcriptome remains unexplored (Sahu et al., 2021).

Reprogramming factors such as Ascl1a, Lin28a, Sox2, Klf4, Myca, Mycb and Pou5f3 (Oct4) are expressed in zebrafish MG following retinal injury (Ramachandran et al., 2010a; Gorsuch et al., 2017; Mitra et al., 2019; Sharma et al., 2019). Forced expression of either Ascl1a and Lin28a, Sox2 or Pou5f3 enhances the regenerative response of MG in the injured zebrafish retina; however, only forced expression of Sox2 is sufficient to stimulate MG proliferation in the uninjured retina (Gorsuch et al., 2017; Elsaeidi et al., 2018; Sharma et al., 2019).

Among the reprogramming factors described above, Myc is unique in that it regulates global RNA levels, interacts with hundreds of proteins, and either directly or indirectly impacts a wide range of cellular processes associated with transcription, RNA processing, protein synthesis, apoptosis, and cell division (Dominguez-Sola et al., 2007; Nie et al., 2012; Conacci-Sorrell et al., 2014; Kalkat et al., 2018; Patange et al., 2022). Because of the role of Myc in stimulating cellular reprogramming and proliferation, it is not too surprising that its expression is associated with tissue regeneration in zebrafish and mammals (Mitra et al., 2019; Illi and Nasi, 2023).

Zebrafish harbor six Myc genes. *myca* and *mycb* are orthologs of mammalian *Myc* (*c-Myc*); *mycla* and *myclb* are orthologs of mammalian *Mycl*; *mycn* is an ortholog of mammalian *Mycn*; and *mych* has no mammalian ortholog (Meijer et al., 2008). Previous studies indicated Myca and Mycb are induced in the injured retina and contribute to MG proliferation (Mitra et al., 2019), and that Mych is necessary for normal retina development (Hong et al., 2008). Whether *mych* and other Myc genes are induced in the injured retina and regulate retina regeneration remains unknown. Furthermore, their contribution to the regeneration-associated transcriptome of MG, their effect on retinal neuron regeneration, as well as their potency in eliciting a regenerative response from MG have never been investigated.

Here, we report that *mycb* and *mych* are the only Myc family members induced in MG following retinal injury. Our studies reveal that Mycb and Mych stimulate MG reprogramming and proliferation, but do not regulate the type of retinal neuron regenerated. Although Myc proteins are often thought to act in a redundant fashion, we identify differences in their contribution to the regeneration-associated transcriptome of MG and in their ability to regulate neuronal apoptosis. We find that Mycb and Mych preferentially regulate the expression of genes controlling cellular processes such as ribosome biogenesis, protein synthesis, DNA synthesis and cell division, which are among the most highly enriched processes in reprogrammed MG, suggesting that Mycb and Mych are potent reprogramming factors. Consistent with this, forced expression of either of these genes is sufficient to stimulate MG proliferation in the uninjured retina, and this is enhanced by γ-secretase inhibition as well as forced expression of Ascl1a and Lin28a. Overall, our studies identify Mycb and Mych as potent reprogramming factors that may be good candidates, alone or in combination with other factors, for stimulating MG reprogramming and retina regeneration in mammals.

## RESULTS

### Injury-dependent regulation of Myc family gene expression

Multiple types of injury have been used to stimulate MG reprogramming and proliferation in the zebrafish retina, including needle poke injury, which ablates all retinal neuron types at the lesion site; intravitreal administration of N-methyl-D-aspartate (NMDA), which ablates amacrine and ganglion cells in the inner nuclear layer (INL) and ganglion cell layer (GCL) of the retina; and intense UV light, which ablates photoreceptors in the outer nuclear layer (ONL) of the retina (Powell et al., 2016; Lyu et al., 2023). Although each injury model may have some unique effects on MG (Lyu et al., 2023), we suspect that the common elements underlying the response of MG to these different types of injuries will reveal a core set of factors that are crucial to stimulation of the MG regenerative response.

**Fig. 1.**
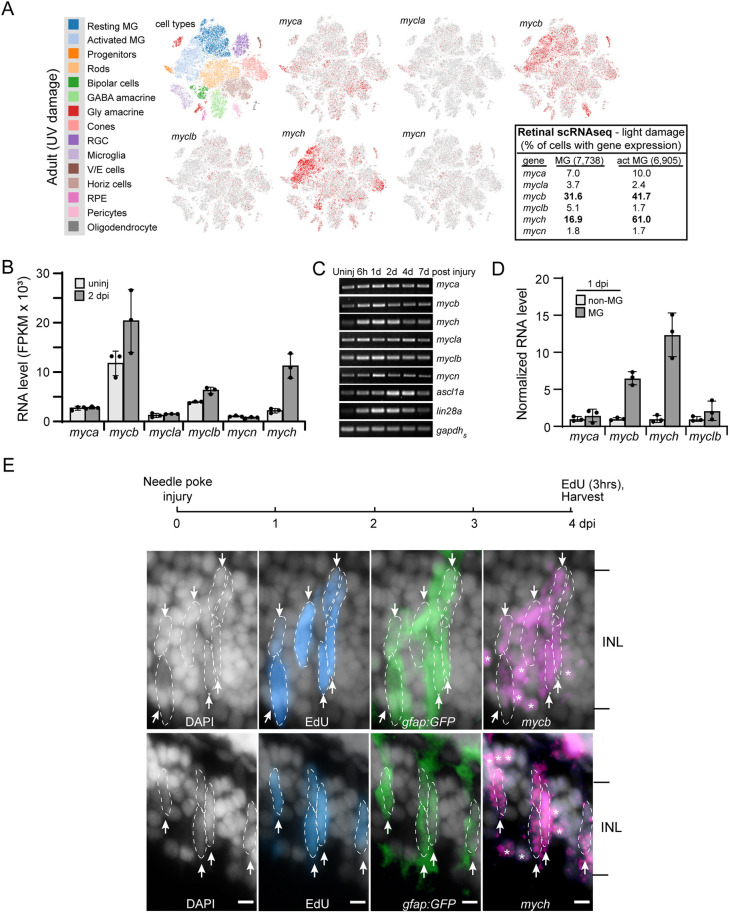
**Myc family gene expression in uninjured and injured zebrafish retina.** (A) Retinal scRNAseq UMAP plots showing Myc family gene expression in uninjured and injured retina (data taken from Hoang et al., 2020). Red dots indicate the Myc gene-expressing cells in the uninjured and injured retina. By comparing resting MG with activated MG one can discern the changes in gene expression as MG respond to retinal injury [the percentage of total and activated (act) MG with detectable expression of each Myc family member is shown in the table shown on the right]. (B) MG RNAseq quantification of normalized gene reads for Myc family members. (C) PCR and agarose gel analysis of temporal changes in Myc family gene expression following retinal injury. (D) RNA levels of select Myc family genes in FACS-separated GFP^+^ MG and GFP^−^ non-MG cells isolated from *gfap:GFP* fish retinas. (E) Top is experimental timeline. *gfap:GFP* fish received a needle poke injury and then an intravitreal injection of EdU 3 h before being euthanized at 4 dpi. Retinal sections were stained with DAPI to identify nuclei (gray/white), click-iT chemistry was used to identify EdU^+^ cells (blue), immunofluorescence was used with anti-GFP antibody to identify MG (green), and fluorescence-based *mycb* and *mych in situ* hybridization assays were used to identify *mycb*- and *mych*-expressing cells (magenta). Arrows and dashed lines outline clusters of *mycb*^+^ or *mych*^+^ MG and MG-derived progenitors that are EdU^+^ and GFP^+^. White asterisks indicate *mycb* and *mych* expression in neurons (*gfap:GFP*^−^ cells). The number of biological replicates (*n*) is indicated by the dots in each graph. act MG, activated Muller glia; d, day; dpi, days post injury; FPKM, fragments per kilobase of transcript per million mapped reads; INL, inner nuclear layer; MG, Müller glia; V/E cells, vascular endothelial cells. Arrows point to EdU^+^;GFP^+^;*mycb*^+^ MG and EdU^+^;GFP^+^;*mych*^+^ MG. Scale bars: 20 μm.

To identify Myc family genes expressed in MG and regulated by retinal injury, we interrogated retinal scRNAseq (UV light injury) and MG RNAseq (needle poke injury) data sets (Hoang et al., 2020; Lee et al., 2020; Sahu et al., 2021). This revealed that *mycb* and *mych* are the only Myc family members induced in activated MG (Fig. 1A,B). However, their expression was not restricted to activated MG as they were also detected in all retinal neuron types (Fig. 1A). RT-PCR assays (needle poke injury) indicate that *mycb* and *mych* are induced within 6 h post-injury (hpi), which corresponds to a time when the regeneration-associated genes *ascl1a* and *lin28a* begin to increase (Fig. 1C).

In *gfap:GFP* transgenic fish, MG, activated MG, and MG-derived progenitors are labeled with GFP (Bernardos and Raymond, 2006; Bernardos et al., 2007). qPCR assays using RNA from fluorescence-activated cell sorting (FACS)-purified GFP^+^ MG and GFP^−^ non-MG from needle poke-injured *gfap:GFP* fish retinas at 1 day post-injury (dpi) confirmed that *mycb* and *mych* are enriched in the activated MG population (Fig. 1D). Furthermore, *mycb* and *mych in situ* hybridization assays combined with 5-ethynyl-2-deoxyuridine (EdU) labeling (to detect cell proliferation) and/or GFP immunofluorescence using *gfap:GFP* fish that received a needle poke injury and an intraperitoneal (IP) injection of EdU 3 h before sacrifice (2-4 dpi) revealed *mycb* and *mych* expression in GFP^+^;EdU^+^ cells residing in the INL (Fig. 1E; Fig. S1A). Because *gfap:GFP* transgenic fish label MG (the predominant glial cell type in the fish retina) and MG-derived progenitors with GFP (Bernardos and Raymond, 2006; Bernardos et al., 2007), and because MG are the only cell type to proliferate in the INL of injured retina (Fausett and Goldman, 2006; Nagashima et al., 2013), the observation that *mycb* and *mych* are expressed in GFP^+^;EdU^+^ cells in the INL confirms their identity as proliferating MG and/or MG-derived progenitors. Consistent with the scRNAseq data, we also observed *mycb* and *mych* expression in GFP^−^ neurons (Fig. 1E, asterisks).

Although a previous study reported injury-dependent induction of *myca* and *mycb* in activated MG (Mitra et al., 2019), our analysis of scRNAseq and MG RNAseq data sets, along with qPCR assays suggest that *myca* is not induced in reprogrammed MG (Fig. 1A-D). Mitra et al. (2019) also reported that *mycb* exhibits a transient pan-retinal expression pattern at 12 hpi that is restricted to activated MG at 2 dpi, yet scRNAseq data sets and *in situ* hybridization assays indicate that *mycb* and *mych* expression persists in retinal neurons even at 4 dpi (Fig. 1A,E).

We were struck by the similar expression profile of *mycb* and *mych* in the uninjured and injured adult light-damaged retina (Fig. 1A) and wondered whether this is also true during development. *In situ* hybridization assays on fish at 1-5 days post-fertilization (dpf) revealed overlapping and unique expression patterns of *mycb* and *mych* genes (Fig. S1B). In the developing eye at 1 dpf, *mycb* was expressed in the lens, whereas *mych* was expressed in the lens and retina (Fig. S1B). At 2 dpf, both genes were detected in the ciliary marginal zone (CMZ) of the retina; however, over the next couple of days, *mycb* became undetectable, whereas *mych* expression persisted (Fig. S1B). Thus, depending on the stage of development and the tissue examined, these genes can have very different expression patterns.

### Conservation of Mycb and Mych proteins with human MYC

scRNAseq data sets from adult retina indicate that *mycb* and *mych* share similar cell type-specific expression patterns (Fig. 1A), perhaps suggesting functional redundancy. However, this redundancy seems less likely when examining MYC homology boxes (MBs), which are highly conserved among all mammalian MYC family members (Conacci-Sorrell et al., 2014; Kalkat et al., 2018). These MBs mediate the interaction of MYC with hundreds of proteins and impact its ability to regulate a variety of processes such as transcription, ribosome biogenesis, and DNA replication (Kalkat et al., 2018).

Overall, zebrafish Mych is 35% identical to Mycb, and Mycb and Mych share 55% and 27% amino acid identity, respectively, with human MYC (Fig. S1C). Most of the differences between Mycb and Mych reside in the amino terminal half of the protein. This region harbors MBs 0-3a, which are involved in transcription and transformation (Kalkat et al., 2018; Lourenco et al., 2021). This region in human MYC shares 60% and 19% amino acid identity with zebrafish Mycb and Mych, respectively. Although MBs 0-3a are poorly conserved in Mych, phospho-accepting residues T74 and S78 in MB1 (indicated by # in Fig. S1C), which regulate MYC activity and stability (Pulverer et al., 1994; Sears et al., 2000; Welcker et al., 2004), remain intact. The MB2 domain interacts with TRRAP-HAT complexes to regulate histone acetylation (Kalkat et al., 2018) and the MB3a domain is involved in inhibition of the apoptotic activity of MYC (Herbst et al., 2005); both of these domains are poorly conserved in Mych. Importantly, it has been reported that deletion of MB0, MB1, MB2 or MB3 results in the loss of 94, 36, 43 or 45 interacting partners, respectively (Kalkat et al., 2018).

The carboxy terminal half of MYC includes MB3b and MB4, along with the basic helix-loop-helix (bHLH) and leucine zipper domains, which are involved in DNA binding and MYC:MAX heterodimerization (Fig. S1C). The MB3b domain of human MYC harbors PEST and WDR5-binding domains, which are conserved in Mycb and Mych. These domains contribute to MYC protein stability and chromatin interaction, respectively (Gregory and Hann, 2000; Thomas et al., 2015). Within the MB4 domain is a calpain cleavage site that is necessary for generating a cytoplasmic form of MYC, called MYC-Nick, that stimulates α-tubulin acetylation and cellular differentiation (Conacci-Sorrell et al., 2010). Although this region is largely conserved between MYC and Mycb, this conservation is less when comparing MYC with Mych. MB4 also contains the HCF-1 binding site, which impacts MYC-driven tumorigenesis by regulating ribosome biogenesis and mitochondrial gene expression (Thomas et al., 2016; Popay et al., 2021), and this sequence is conserved in both Mycb and Mych.

The above sequence comparisons indicate that Mych is the most divergent Myc family member and that several MBs conserved between human MYC and zebrafish Mycb are not conserved in Mych. This divergence in protein sequence may indicate functional differences in these proteins and warrants further comparison of Mycb and Mych function during retina regeneration.

### Mycb and Mych stimulate proliferation of MG and MG-derived progenitors in the injured retina

**Fig. 2.**
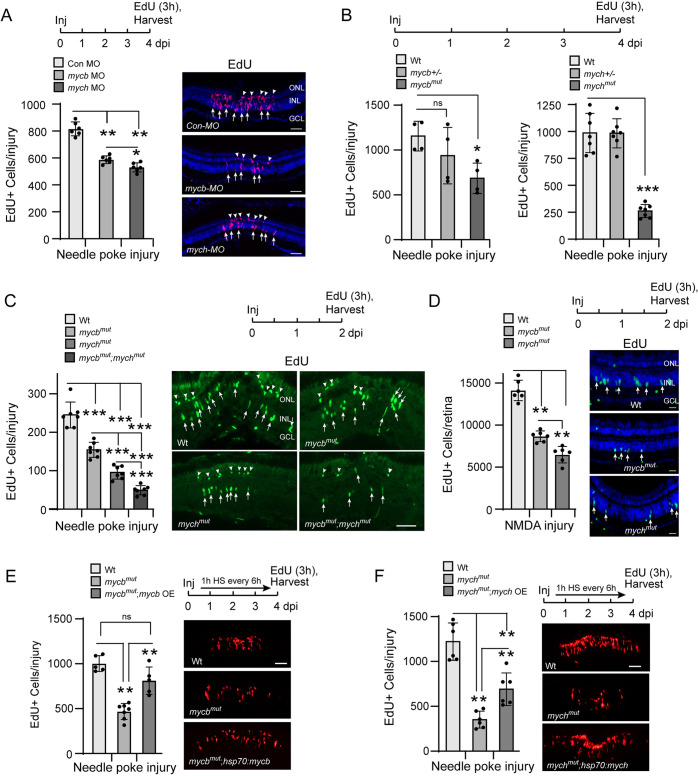
**Mycb and Mych regulate MG proliferation in the injured retina.** (A) Experimental timeline is shown at the top. Bar graph (left) and representative images (right) show the consequence of Mycb and Mych knockdown with antisense morpholino-modified oligonucleotide (MO) on MG proliferation (EdU^+^ cells, magenta, arrows) and rod progenitor proliferation (arrowheads in ONL) in needle poke-injured retina at 4 dpi. Retinal cells identified with DAPI (blue). (B) Experimental timeline is shown at the top. Bar graphs show the consequence of Mycb and Mych mutation on MG proliferation (EdU^+^ cells) in needle poke-injured retina at 4 dpi. (C) Experimental timeline is shown above the photomicrographs (right). Arrows point to proliferating MG in the INL and arrowheads point to proliferating rod progenitors in the ONL. Bar graph (left) and representative images (right) show the consequence of individual and combined Mycb and Mych mutation on MG proliferation (EdU^+^ cells; green) in the needle poke-injured retina at 2 dpi. (D) As in C, but NMDA was used to injure the retina. Retinal cells identified with DAPI (blue). (E,F) Experimental timeline is shown above the photomicrographs [arrow indicates 1 h heat shock (HS) repeated every 6 h]. Bar graph (left) and representative images (right) show that *mycb* OE rescues MG proliferation (EdU detection, red) in *mycb^mut^;hsp70l:mycb* fish (E), and *mych* OE only partially rescues MG proliferation in *mych^mut^;hsp70l:mych* fish (F). The number of biological replicates (*n*) is indicated by the dots in each graph. Error bars are s.d. *P<0.05, **P<0.01, ***P<0.001. dpi, days post-injury; GCL, ganglion cell layer; HS, heat shock; Inj, injury; INL, inner nuclear layer; ns, not significant; OE, overexpression; ONL, outer nuclear layer. Scale bars: 100 μm (A,C,E,F); 50 μm (D).

Myc family gene expression is often associated with cell proliferation and tumor formation. Retinal injury stimulates proliferation of MG and MG-derived progenitors that are identified as clusters of two or more EdU^+^ cells in the INL (Fausett and Goldman, 2006; Nagashima et al., 2013). To investigate whether Mycb and Mych stimulate injury-dependent MG proliferation in the zebrafish retina, we knocked down their expression using morpholino modified antisense oligonucleotides (MOs). Fish retinas were injured with a needle poke injury and then a control or an experimental MO was electroporated into the retina as previously described (Fausett and Goldman, 2006; Fausett et al., 2008). Fish received an IP injection of EdU 3 h before being euthanized at 4 dpi. Quantification of EdU^+^ cells indicated reduced MG proliferation following either Mycb or Mych knockdown (Fig. 2A). The Mycb knockdown effect on MG proliferation was similar to that reported previously (Mitra et al., 2019).

We confirmed these results using F0 CRISPR gene-edited fish. For this, zebrafish embryos injected with Cas9-nanos-encoding mRNA and two different gRNAs designed to generate a deletion in either the *mycb* or *mych* gene (*mycb*-gRNA_1,2_ and *mych*-gRNA_1,2_ in Fig. S2A). PCR confirmed *mycb* and *mych* gene editing (Fig. S2B), and a needle poke injury to adult F0 fish retinas confirmed that gene-edited fish exhibit reduced proliferation of MG-derived progenitors (Fig. S2C,D).

Although the above data suggested that Mycb and Mych suppression have a similar effect on injury-dependent MG proliferation, we were concerned that incomplete inactivation of these genes in MO-electroporated retinas and the mosaic nature of gene editing in F0 fish may hinder our ability to detect differences in Mycb and Mych activity. Therefore, we created new zebrafish lines harboring germline mutations in the *mycb* and *mych* genes. For this, embryos were injected with either two different *mycb*-gRNAs targeting exon 2 (*mycb*-gRNA_1,2_) or a single *mych*-gRNA (*mych*-gRNA_1_) targeting exon 2, along with *Cas9-nanos* mRNA (Fig. S2E,H). F0 injected fish with indels were identified and bred with wild-type (Wt) fish (Fig. S2F,I). Fish with heterozygous mutations were inbred to generate homozygous mutants. DNA sequencing identified a *mycb* indel with a 197 bp deletion after codon 64 that changes the reading frame and adds 36 new amino acid codons followed by a stop codon (Fig. S2G), whereas the *mych* indel was a 6 bp deletion with a 5 bp insertion resulting in a reading frame shift at codon 35 that adds nine new amino acid codons followed by a stop codon (Fig. S2J). In addition, a novel NlaIII restriction site was generated that facilitated mutant identification (Fig. S2I,J). Homozygous *mycb* and *mych* mutant fish were generated at normal Mendelian ratios. In contrast to a previous report indicating that Mych expression is necessary for normal retina lamination and development (Hong et al., 2008), we did not observe any obvious defects in lamination or MG differentiation in developing retinas of *mycb^mut^* and *mych^mut^* fish (Fig. S2K,L). We suspect that the developmental defects noted by Hong et al. (2008) might be due to *mych*-MO off-target effects.

Consistent with the above-described MO knockdown and F0 CRISPR-edited fish, injury-dependent (needle poke) MG proliferation was reduced in *mycb^mut^* and *mych^mut^* fish when assayed at 2, 4 or 6 dpi, with proliferation suppressed the most in *mych^mut^* fish (Fig. 2B,C; Fig. S2M,N). Furthermore, regardless of the type of injury (needle poke or NMDA), Mycb and Mych were observed to contribute to MG proliferation (Fig. 2C,D).

To investigate whether loss of Mycb and Mych function impacts neural regeneration in a cell type-specific manner, we injured Wt or *mycb^mut^;mych^mut^* retinas with a needle poke and labeled proliferating MG at 4 dpi with EdU. At 30 dpi, fish were euthanized, and retinal sections were assayed for EdU^+^ retinal neurons. Quantification of total EdU^+^ cells and EdU^+^ cells that co-stained with the neuronal markers Zpr1 (photoreceptors), Pkc_α_ (bipolar neurons), and HuC/D (amacrine and ganglion neurons) revealed a reduction in total EdU^+^ neurons in double mutants compared with Wt (∼43%) (Fig. S2O), suggesting a permanent impairment of progenitor proliferation. However, the percentage of EdU^+^ cells regenerating specific retinal neuron types in mutant fish remained similar to that found in Wt fish (Fig. S2P), and this suggests that progenitor differentiation is unaffected by *mycb* and *mych* mutations.

Although *mycb^mut^* and *mych^mut^* fish appeared normal, double mutants were thinner than individual mutants or Wt fish (Fig. S2Q). We also noted a significant reduction in body weight in single and double mutant fish at 4 months of age, whereas body length was only slightly reduced in *mycb^mut^;mych^mut^* fish (Fig. S2R,S). This prompted us to consider whether loss of Mycb or Mych function during development might impact adult retinal biology in such a way that regeneration is perturbed. To investigate this, we attempted to rescue the reduced proliferation phenotype noted in *mycb^mut^* and *mych^mut^* fish by overexpressing Mycb and Mych, respectively. For this, we generated *hsp70l:mycb;cmlc:GFP* (also called *hsp70l:mycb*) and *hsp70l:mych;cmlc:GFP* (also called *hsp70l:mych*) transgenic fish that allow for conditional Myc gene expression with heat shock. Although not quantified, we noticed a small number of fish with cardiac tumors/cardiac hypertrophy in the *hsp70l:mycb;cmlc:GFP* and *hsp70l:mych;cmlc:GFP* transgenic fish that was reflected by increased expression of the cardiac marker gene *cmlc:GFP* (representative tumor-bearing *hsp70l:mych;cmlc:GFP* fish in Fig. S3A). This suggests some leakiness in transgene expression. Fish with visible tumors were not used in our study. RT-PCR and *in situ* hybridization assays confirmed that a single 1 h heat-shock treatment at 37°C increased the expression of *mycb* and *mych* (Fig. S3B,C).

*hsp70l:mycb* and *hsp70l:mych* fish were bred with *mycb^mut^* and *mych^mut^* fish, respectively. *hsp70l:mycb;mycb^mut^* or *hsp70l:mych;mych^mut^* fish retinas were injured with a needle poke and Myc expression was induced with heat shock. At 4 dpi, fish received an IP injection of EdU 3 h before being euthanized. Forced overexpression (OE) of Mycb almost completely rescued MG proliferation in *mycb^mut^* fish, whereas Mych OE only partially rescued MG proliferation in *mych^mut^* fish (Fig. 2E,F). This latter result may indicate that early loss of Mych function can have a lasting impact on retina regeneration. However, we have not ruled out the possibility that this partial rescue is a result of reduced Mych protein expression compared with Mycb. Nonetheless, together with MO-mediated acute Mycb and Mych knockdown in adult fish (Fig. 2A), the above data suggest that Mycb and Mych collaborate to regulate MG proliferation in the injured retina.

### Mycb and Mych OE stimulates proliferation of quiescent MG in the injured dorsal retina

**Fig. 3.**
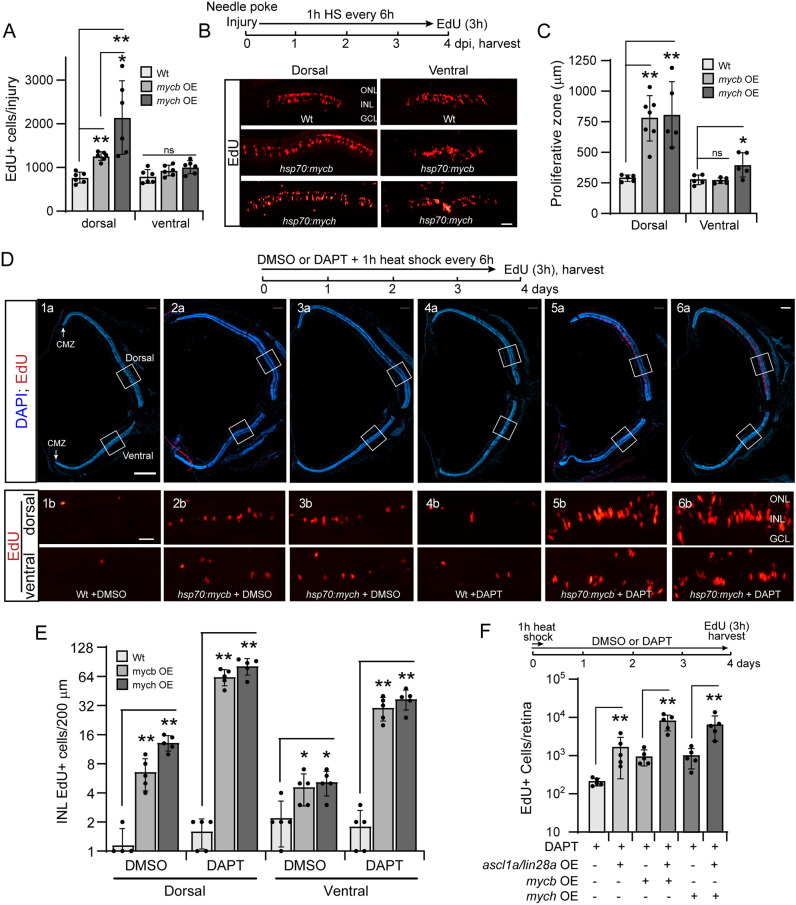
**Mycb and Mych OE stimulate MG proliferation in the injured and uninjured retina.** (A) Bar graph indicating increased MG proliferation (EdU detection, red) at 4 dpi in dorsal and ventral needle poke injured retina with or without Mycb or Mych OE (1 h heat shock repeated every 6 h). Experimental timeline is shown in B. (B) Top: Experimental timeline. Bottom: Representative images used to quantify EdU^+^ cells as shown in A. (C) Bar graph showing the width of the zone of proliferating MG in the needle poke-injured retina with Mycb or Mych OE as described in A. (D) Experimental timeline is shown at the top. Panels 1a-6a show whole uninjured retina sections taken near the optic nerve head. DAPI-stained nuclei are blue and EdU^+^ cells are red. Wt and transgenic fish ±DAPT were treated with 1 h heat shock every 6 h, and 3 h before being euthanized fish received an IP injection of EdU. White squares are examples of the dorsal and ventral retinal regions magnified in panels 1b-6b and used for the quantification shown in E. (E) Bar graph showing quantification of EdU^+^ cells in uninjured retinas treated as in D. (F) Bar graph showing the effects Mycb and Mych OE (1 h heat shock every 6 h) have on MG proliferation (EdU^+^ cells) in DAPT-treated uninjured retinas with and without Ascl1a/Lin28a OE (1 h heat shock every 6 h). Fish received an IP injection of EdU 3 h before sacrifice on day 4. The number of biological replicates is indicated by the dots in each graph. Error bars are s.d. *P<0.05, **P<0.01. The number of biological replicates (*n*) is indicated by the dots in each graph. dpi, days post-injury; GCL, ganglion cell layer; HS, heat shock; INL, inner nuclear layer; IP, intraperitoneal; ns, not significant; OE, overexpression; ONL, outer nuclear layer. Scale bars: 50 μm (B); 100 μm (D).

Although the above data suggested that forced expression of Mycb and Mych stimulates proliferation of MG and MG-derived progenitors in the injured retina, the dorsal retina appeared to be responding to this forced expression more than ventral retina. To investigate this further, *hsp70l:mycb* and *hsp70l:mych* transgenic fish received a single needle poke injury to either the dorsal or ventral retina and the number of EdU^+^ cells in the INL was quantified. In addition, the width of the zone of proliferating MG surrounding the injury site was measured to determine whether more MG were recruited to an injury response with Mycb and Mych OE. In the injured dorsal retina, either Mycb or Mych OE increased the number of EdU^+^ cells in the INL and this was reflected by an expanded zone of injury-responsive MG surrounding the injury site (Fig. 3A-C). In the injured ventral retina, there was a small expansion in the zone of proliferating MG in Mych OE fish; however, this was not accompanied by a significant increase in the number of proliferating MG (Fig. 3A-C). The enhanced MG proliferation noted in the dorsal retina of *hsp70l:mycb* or *hsp70l:mych* overexpressing fish was not due to dorsal biases in transgene expression and was not observed in *mycb^mut^* and *mych^mut^* fish (Fig. S3C-E).

We previously reported that γ-secretase inhibition with DAPT expanded the zone of proliferating MG in the injured retina and attributed it to a lowering of MG's injury response threshold (Wan et al., 2012; Sahu et al., 2021). The above data suggest that Mycb and Mych may also contribute to the injury response threshold of MG. Therefore, we wondered whether Myc OE might synergize with γ-secretase inhibition to stimulate MG proliferation in the uninjured retina. Before examining their combined effect, we first assayed MG proliferation in uninjured Wt, *hsp70l:mycb* and *hsp70l:mych* fish treated with a 1 h heat shock at 37°C every 6 h for 4 days. Three hours before being euthanized, fish received an IP injection of EdU. Quantification of EdU^+^ MG in the INL revealed ∼7- and ∼13-fold increases in MG proliferation in uninjured dorsal retina of *hsp70l:mycb* and *hsp70l:mych* fish, respectively (Fig. 3D1a-D3b,E). An increase in proliferation of putative rod progenitors, identified by their round shape and their restriction to the ONL, was also noted in these fish (Fig. S3F).

Although γ-secretase treatment alone had little effect on MG proliferation in the uninjured retina, when combined with Mycb or Mych OE, MG proliferation increased ∼40- and ∼51-fold, respectively, in the dorsal retina (Fig. 3D4a-D6b,E), and this exceeded MG proliferation in the ventral retina by ∼1.4- and 2.2-fold, respectively (Fig. 3E). Co-staining retinal sections for EdU and glutamine synthetase (GS) confirmed that proliferating cells in INL were MG and MG-derived progenitors (Fig. S3G). It is notable that these conditions for stimulating MG proliferation in the uninjured retina did not stimulate proliferation of retinal progenitors residing in the CMZ, which are responsible for retinal expansion throughout the fish's life (Fig. 3D).

We previously reported that treating uninjured retina with DAPT along with Ascl1 and Lin28a OE was sufficient to stimulate MG proliferation in the uninjured retina (Elsaeidi et al., 2018). Here, we investigated whether Mycb or Mych OE enhanced this proliferative response. For this analysis, we bred our *hsp70l:mycb* and *hsp70l:mych* fish individually with *hsp70l:ascl1a;hsp70l:lin28a* double transgenic fish. Transgenic fish received a single 1 h heat-shock treatment at the beginning of the study and were then immersed in DAPT-treated fish water for 4 days before assaying MG proliferation with a pulse of EdU 3 h before being euthanized. Remarkably, even with a single 1 h heat-shock treatment, Mycb and Mych OE greatly enhanced the proliferation observed with the combinatorial treatment of DAPT and forced expression of Ascl1a and Lin28a (Fig. 3F; Fig. S3H).

Together, the above data indicate that Mycb or Mych OE is sufficient to stimulate MG proliferation in the uninjured retina, but when combined with γ-secretase inhibition and/or Ascl1a/Lin28a OE a much stronger proliferative response is observed.

### Regulation of neuron apoptosis by Mycb and Mych

MYC stimulates apoptosis under certain conditions (McMahon, 2014), and apoptotic neurons are a stimulus for engaging MG in a regenerative response (Vihtelic and Hyde, 2000; Bailey et al., 2010; Montgomery et al., 2010; Nelson et al., 2013; Mitra et al., 2022). Therefore, we wondered whether Mycb and/or Mych apoptotic activity contributed to their ability to stimulate MG proliferation in the injured retina. NMDA was previously shown to stimulate amacrine and ganglion cell death in the INL and GCL of the retina, with maximal apoptosis detected at 1 dpi (Powell et al., 2016). In addition to retinal neurons, zebrafish harbor microglial cells and MG in their retina. To investigate the cell types dying in response to NMDA-induced retinal injury (1 dpi), we stained retinal sections with HuC/D to detect amacrine and ganglion cell neurons, 4C4 to detect microglial cells, and terminal deoxynucleotidyl transferase dUTP nick end labeling (TUNEL) to detect apoptotic cells. As expected, the only TUNEL^+^ cells were amacrine and ganglion cells residing in the INL and GCL (Fig. S4A).

**Fig. 4.**
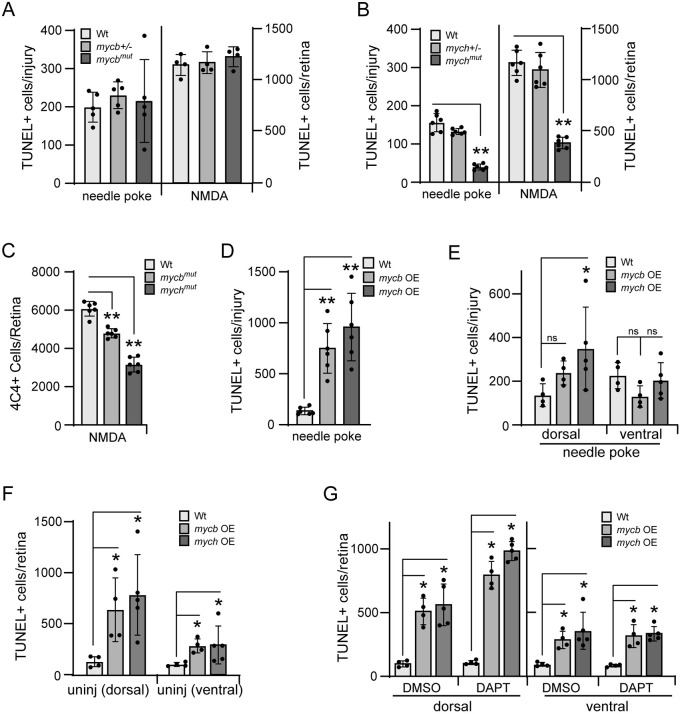
**Regulation of neuronal apoptosis by Mycb and Mych in injured and uninjured retinas.** (A,B) Bar graphs showing the number of TUNEL^+^ cells in needle poke and NMDA-treated retinas of Wt (A,B), *mycb^mut^* (A) and *mych^mut^* (B) fish at 1 dpi. (C) Bar graph showing the number of 4C4-positive cells in Wt, *mycb^mut^* and *mych^mut^* fish at 2 dpi. (D) Bar graph showing the number of TUNEL^+^ cells in needle poke-injured fish retina overexpressing Mycb or Mych at 1 dpi. (E,F) Bar graphs showing the number of TUNEL^+^ cells in dorsal and ventral regions of injured (E) or uninjured (F) retinas with Mycb or Mych OE. (G) Bar graph showing the number of TUNEL^+^ cells in dorsal and ventral regions of uninjured retinas treated with DAPT and Mycb or Mych OE for 1 day. The number of biological replicates (*n*) is indicated by the dots in each graph. Error bars are s.d. *P<0.05, **P<0.01. The number of biological replicates is indicated by the dots in each graph. OE, overexpression.

We then investigated whether Mycb and/or Mych apoptotic activity contributed to neuron death and MG proliferation in the injured retina. For this, we assayed TUNEL^+^ cells in Wt, *mycb^mut^* and *mych^mut^* fish at 1 dpi. Interestingly, the number of TUNEL^+^ cells was similar in injured (needle poke or NMDA-treated) retinas from Wt and *mycb^mut^* fish (Fig. 4A; Fig. S4B), whereas TUNEL^+^ cells were reduced in injured retinas of *mych^mut^* fish (Fig. 4B; Fig. S4C). Although *mycb^mut^* fish had normal numbers of TUNEL^+^ cells in injured retinas (Fig. 4A), EdU^+^ cells were reduced (Fig. 2D). These TUNEL^+^ neurons are thought to initiate MG reprogramming via direct communication with MG, but dying neurons also attract microglial cells to the injury site and these cells are the predominant immune cell type found in the injured retina (White et al., 2017). Microglia also appear to stimulate a second reprogramming event that is necessary for MG proliferation (Mitra et al., 2022). Therefore, we wondered whether immune cell numbers at sites of retinal injury might better reflect MG proliferation than TUNEL^+^ neurons. Indeed, 4C4 staining of microglia in NMDA-injured retinas from Wt, *mycb^mut^* and *mych^mut^* fish revealed reduced 4C4^+^ microglia in *mycb^mut^* fish that were reduced even further in *mych^mut^* fish (Fig. 4C; Fig. S4D).

Consistent with the intrinsic apoptotic activity emerging when Myc is expressed at high levels, we found that either Mycb or Mych OE in the uninjured or needle poke-injured retina for 1 day increased the number of TUNEL^+^ cells (Fig. 4D-G). Importantly, this Myc-dependent stimulation of apoptosis exhibited a dorsal bias (Fig. 4E,F) which was enhanced in DAPT-treated fish (Fig. 4G; Fig. S4E).

Together, these data suggest that the intrinsic apoptotic activity of Myc is most readily apparent when Myc proteins are overexpressed, and it seems likely that this intrinsic activity collaborates with the dorsal environment of the retina to further enhance apoptosis. However, in the injured retina, only endogenous Mych apoptotic activity is sufficient to stimulate neuron death, and this may contribute to its ability to enhance MG proliferation over that of Mycb.

### Mycb and Mych contributions to the regeneration-associated transcriptome of MG

The regeneration-associated transcriptome of MG comprises genes for which expression is regulated by retinal injury (Fig. S5A, left plot). Because of the potent transcriptional and reprogramming activities of Myc, it has the potential to contribute disproportionately to the regeneration-associated transcriptome of MG. To identify these contributions, we compared the regeneration-associated transcriptome of MG from Wt (*gfap:GFP*) fish with that of *gfap:GFP;mycb^mut^* and *gfap:GFP;mych^mut^* fish using FACS-purified GFP^+^ MG for RNAseq (Fig. S5A). Regeneration-associated genes from Wt fish that were dysregulated in either *mycb^mut^* and/or *mych^mut^* fish are referred to as Mycb- and/or Mych-regulated regeneration-associated genes. This analysis showed that ∼40% of the genes comprising the MG regeneration-associated transcriptome was regulated by Mycb, whereas only ∼16% was regulated by Mych.

**Fig. 5.**
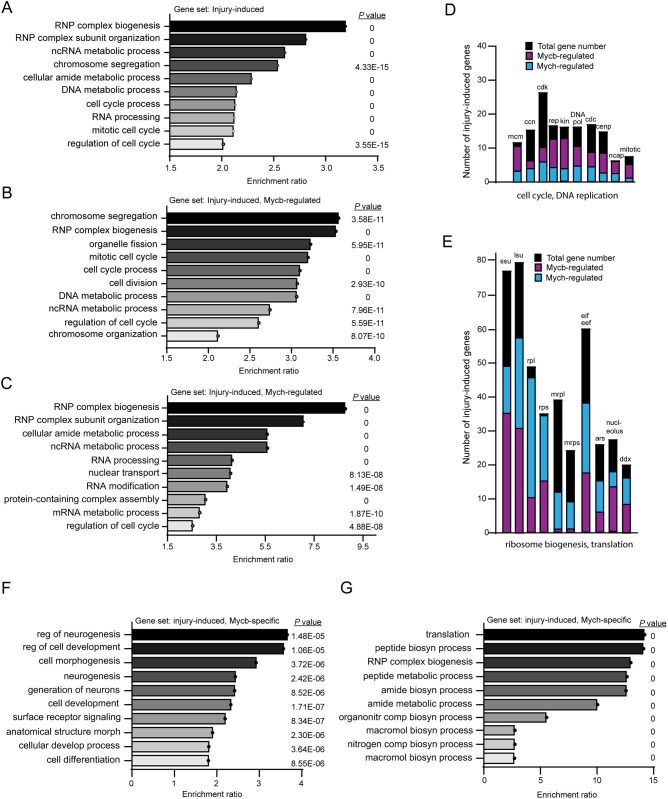
**Cellular process enrichment analysis reveals that Mycb and Mych regulate biological processes associated with ribosome biogenesis, protein synthesis, chromosome segregation, and cell cycle.** (A-C,F,G) Top 10 enriched biological processes for injury-induced genes in Wt fish (A), Mycb-dependent injury-induced genes (B), Mych-dependent injury-induced genes (C), Mycb-, but not Mych-dependent injury-induced genes (F), and Mych-, but not Mycb-dependent injury-induced genes (G). (D,E) Examples of gene families that are induced in reprogrammed MG and the number of family members that are regulated by Mycb and/or Mych. ars, aminoacyl tRNA synthetases; ccn, cyclin; cdc, cell division cycle; cdk, cyclin-dependent kinase; cenp, centromere protein; ddx, deadbox genes; DNA pol, DNA polymerase; eif eef, eukaryotic translation initiation factors and eukaryotic translation elongation factors; kin, kinetochore; lsu, large ribosomal subunit biogenesis; mcm, minichromosome maintenance; mrpl, mitochondrial ribosomal protein large; mrps, mitochondrial ribosomal protein small; ncap, non-SMC condensing; rep, DNA replication; rpl, ribosomal protein large; rps, ribosomal protein small; ssu, small ribosome subunit biogenesis.

To identify cellular processes that are stimulated in the injured retina by Mycb and/or Mych, we used WebGestalt, a web-based functional enrichment tool (Liao et al., 2019). We first assayed for enrichment of biological processes in response to genes induced by retinal injury (Fig. 5A). Among the top 10 enriched processes were ‘ribonucleoprotein (RNP) complex biogenesis’, ‘ncRNA metabolic processes’, ‘chromosome segregation’, ‘cellular amide metabolic processes’, ‘DNA metabolic processes’, ‘cell cycle’ and ‘RNA processing’. We then did a similar analysis for Mycb- and/or Mych-regulated regeneration-associated genes that are normally induced in the injured retina. Interestingly, we found that the top Mycb-regulated process include eight of the top 10 processes induced by retinal injury, and the top Mych-regulated processes include five of the top 10 processes induced by retinal injury (Fig. 5A-C). Thus, Mycb- and Mych-regulated genes that are induced in the injured retina disproportionately contribute to the top cellular processes induced in MG during retina regeneration. Furthermore, this functional enrichment analysis suggested biases in the cellular processes regulated by Mycb and Mych, with cell cycle-related processes impacted more by Mycb and protein synthesis-related processes impacted more by Mych (Fig. 5B,C). Indeed, when we quantified the number of injury-induced genes associated with these cellular processes and regulated by Mycb and/or Mych, we found that more Mycb-regulated genes contribute to the cell cycle and DNA replication than Mych-regulated genes, whereas more Mych-regulated genes contribute to ribosome biogenesis and RNA translation processes than Mycb-regulated genes (Fig. 5D,E; Fig. S5B-D; Tables S2, S3).

We next used intersectional analysis to identify genes that are specifically regulated by either Mycb or Mych (Tables S4, S5). For this, we compared the log2 fold difference of injury-responsive genes in MG from Wt fish with those from either *mycb^mut^* or *mych^mut^* fish. Log2 fold changes between −0.5 and 0.5 were considered insignificant. This analysis revealed 1182 injury-responsive genes that were specifically regulated by Mycb, and 436 injury-responsive genes that were specifically regulated by Mych. Using the WebGestalt functional enrichment analysis tool, we found that genes specifically induced by Mycb, but not Mych, were enriched for cellular processes associated with neurogenesis and regulation of cell development, whereas genes specifically induced by Mych, but not Mycb, were enriched for cellular processes associated with protein synthesis and RNP complex biogenesis (Fig. 5F,G; Tables S4, S5). Some of the gene expression changes identified by RNAseq and described above were validated by qPCR (Fig. S5E,F).

Taken together, the above data reveal common and unique contributions that Mycb and Mych make to the regeneration-associated transcriptome of MG. Importantly, these genes preferentially induce the same cellular processes that are most highly enriched in injury-responsive MG and this likely underlies their potency in stimulating MG reprogramming and proliferation in the uninjured retina.

### Mycb and Mych regulate cell division and protein synthesis in reprogrammed MG

**Fig. 6.**
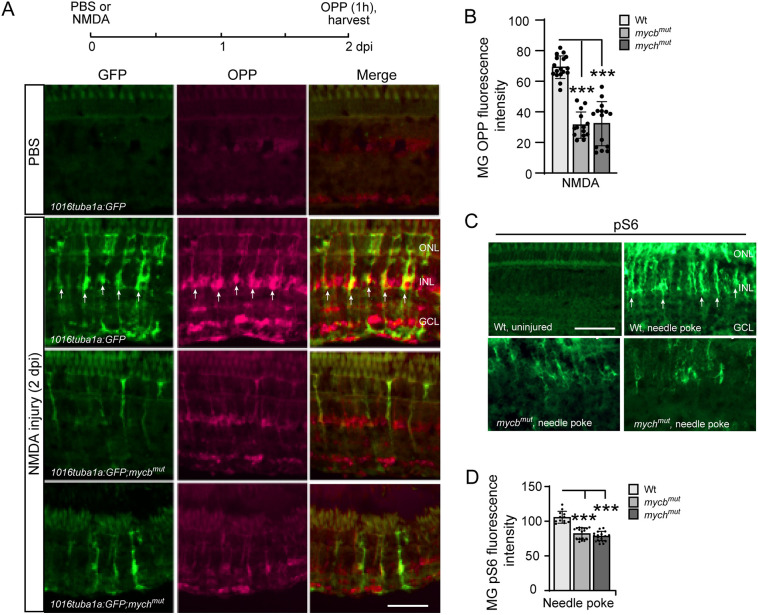
**Injury-induced protein synthesis in reprogrammed MG is regulated by Mycb and Mych.** (A) Experimental timeline is shown at the top. Representative images showing OPP-based protein synthesis detection in *1016 tuba1a:GFP*, *1016 tuba1a:GFP;mycb^mut^* and *1016 tuba1a:GFP;mych^mut^* fish retinas at 2 days post-intravitreal injection of PBS or NMDA. Arrows point to GFP^+^/OPP^+^ co-stained cells, which are predominantly detected in *1016 tuba1a:GFP* fish. (B) Quantification of data presented in A and additional experiments. (C) pS6 immunofluorescence on uninjured and injured (2 dpi) retinas from Wt, *mycb^mut^* and *mych^mut^* fish. Arrows point to representative pS6^+^ cells, which are predominantly detected in the injured fish retina. (D) Quantification of pS6 immunofluorescence in Wt, *mycb^mut^* and *mych^mut^* fish. The number of biological replicates (*n*) is indicated by the dots in each graph. ***P<0.001. dpi, days post injury; GCL, ganglion cell layer; INL, inner nuclear layer; ONL, outer nuclear layer; OPP, O-propargyl-puromycin. Scale bars: 100 μm.

The above data indicated that Mycb and Mych regulate genes associated with MG cell division and protein synthesis (Fig. 5B-E). To investigate this further, we took advantage of *1016 tuba1a:GFP* transgenic fish, which report MG cell division with increased GFP expression (Fausett and Goldman, 2006). Transgenic fish retinas were injured with an intravitreal injection of PBS or NMDA, and at 2 dpi fish received an intravitreal injection of O-propargyl-puromycin (OPP), which is incorporated into nascent polypeptides and can be detected by click-iT chemistry (Liu et al., 2012). One hour after OPP injection, fish were euthanized, and retinal sections processed for GFP immunofluorescence and fluorescence-based detection of OPP-containing polypeptides. This analysis revealed a strong increase in GFP fluorescence and OPP-containing polypeptides in reprogrammed MG (Fig. 6A). Importantly, this injury-dependent increase in MG proliferation and protein synthesis was suppressed in *mycb^mut^* and *mych^mut^* fish (Fig. 6A,B).

mTOR is a serine/threonine kinase the function of which is to integrate a variety of extracellular and intracellular signals to regulate cell growth and proliferation (Laplante and Sabatini, 2012). Among the various cellular processes regulated by mTOR signaling is protein synthesis, during which mTOR regulates the activity of S6 kinase, which subsequently phosphorylates ribosomal protein S6 (Rps6) (Laplante and Sabatini, 2012). Thus, S6 phosphorylation can serve as a readout of mTOR activity. mTOR/pS6 signaling is stimulated in MG following retinal injury and is necessary for MG proliferation (Zhang et al., 2019). Consistent with Mycb and Mych regulating protein synthesis, we found that the injury-dependent (NMDA) increase in pS6 immunofluorescence is suppressed in injured retinas of *mycb^mut^* and *mych^mut^* fish (Fig. 6C,D).

## DISCUSSION

The major findings from this study are: (1) Mycb and Mych stimulate MG reprogramming and proliferation, but do not regulate the specific type of retinal neuron regenerated in the injured retina; (2) Mych exhibits a higher intrinsic apoptotic activity than Mycb, and this correlates with its enhanced regenerative properties; (3) Mycb and Mych regulate ∼40% and 16%, respectively, of the regeneration-associated transcriptome and this is biased towards regulating cellular processes such as protein synthesis and cell division, which are the major processes enriched in reprogrammed MG; (4) although Mycb and Mych regulate many of the same genes, they also exhibit biases, suggesting non-redundant functions, exemplified by their different intrinsic apoptotic activities and the cellular processes they preferentially regulate; and (5) Mycb and Mych are strong reprogramming factors that stimulate MG reprogramming and proliferation in the uninjured retina, and this is enhanced by γ-secretase inhibition and Ascl1a/Lin28a OE.

Although basal expression of *mycb* and *mych* is detected in all retinal neurons and MG, *mycb* exhibits a relatively high basal expression in MG. Interestingly, this expression does not stimulate cellular transformation or tumor formation. However, this expression may contribute to the small amount of spontaneous MG proliferation noted in the uninjured retina that is responsible for producing rod progenitors involved in retinal expansion (Raymond et al., 1988; Bernardos et al., 2007).

Retinal injury stimulates *mych* expression, and, to a lesser extent, *mycb* expression in MG that reprogram for retinal repair. Based on their similar expression pattern in the retina, one might think that Mycb and Mych act redundantly to stimulate injury-dependent MG reprogramming. However, amino acid sequence comparison among the various Myc family members suggests that Mych is the most divergent member of this family. For example, Myca and Mycb share ∼80% amino acid identity, whereas Mych and Mycb share only ∼35% amino acid identity. Furthermore, Mych is the only zebrafish Myc family member that does not have a mammalian ortholog. Thus, we suspect that the different effects these two proteins have on MG reprogramming, proliferation, and neuronal apoptosis would be reflected by differences in their amino acid sequence. Interestingly, the Myc amino terminal transactivation domain regulates its transcriptional and apoptotic activity, and this domain is poorly conserved between Mycb and Mych. This domain harbors several lysine residues that are ubiquitylated to enhance the transcriptional activity of Myc and suppress its apoptotic activity (Zhang et al., 2013). Importantly, these lysine residues are conserved in Mycb, but not in Mych, and might underlie their different transcriptional and apoptotic activities.

It is interesting that the intrinsic apoptotic activity of Mych is reflected in neuronal apoptosis but not in MG apoptosis where this gene is most highly induced. Although the reason for this is not known, MG appear to be a hardy cell type that is able to resist deadly cellular onslaughts such as ouabain treatment (Fimbel et al., 2007; Sherpa et al., 2008). Although we looked for gene expression signatures that may underlie this anti-apoptotic response by MG, none was readily apparent. The mechanism underlying Mych-dependent neuronal apoptosis remains unknown; however, it might result from Mych in neurons impacting apoptotic-related gene expression programs, or, alternatively, Mych in MG may regulate expression of secreted factors that impact apoptosis in neighboring neurons. Further work is needed to distinguish these possibilities.

Although endogenous expression of Mych is sufficient to reveal its pro-apoptotic activity, this activity emerges in both Mycb and Mych when they are overexpressed. This result differs from those reported during zebrafish development where Mych suppresses neuronal apoptosis (Kotkamp et al., 2014), yet it is consistent with previous observations indicating that Myc expression levels dictate its biological output, with lower Myc levels preferentially driving cell proliferation and higher levels revealing its intrinsic apoptotic activity (Murphy et al., 2008). This enhanced apoptosis with either Mycb or Mych OE was found to synergize with the γ-secretase inhibitor DAPT to further stimulate neuronal apoptosis and MG proliferation. A similar synergy was reported for γ-secretase-treated neuroblastoma cells that overexpress MYCN, and this was attributed, at least in part, to increased expression of the pro-apoptotic protein NOXA (Dorneburg et al., 2016).

As the initial stimulus for engaging MG in a regenerative response is dying neurons (Vihtelic and Hyde, 2000; Fimbel et al., 2007; Montgomery et al., 2010; Mitra et al., 2022), we suspect that the increased neuronal apoptosis associated with endogenous Mych expression and the forced expression of either Mycb or Mych will result in more microglia being recruited to injury sites where they will then enhance MG proliferation. In addition, we previously reported that γ-secretase inhibition or Notch signaling inhibition with dominant-negative MAML, lowers the MG injury-response threshold (Sahu et al., 2021), and this too may contribute to the noted synergy when Mycb or Mych is overexpressed in the presence of γ-secretase inhibitors.

We noted that forced expression of either Mycb or Mych stimulated more neuronal apoptosis and MG proliferation in the dorsal retina than in the ventral retina. This was not due to differences in transgenic RNA expression along the dorsal-ventral axis and may reflect different dorsal/ventral environments of the retina that are determined by morphogenetic gradients (Kishimoto et al., 1997; Gosse and Baier, 2009). However, we cannot rule out that this reflects different levels of Mycb and Mych protein expression.

To identify biological processes activated by gene expression programs induced in reprogrammed MG, we performed a gene ontology term enrichment analysis using the injury-induced genes that comprise the MG regeneration-associated transcriptome. Among the top 10 biological processes enriched in injury-responsive MG are those related to ribosome biogenesis, protein synthesis, DNA synthesis, and cell division. These biological processes are at the heart of stem cell reprogramming as they prepare a cell for making large amounts of protein and DNA needed for cell division. Genes controlling these retinal stem cell-related biological processes are likely to be important reprogramming factors that may be good candidates for stimulating a regenerative response by mammalian MG. Consistent with this, we found that Mycb and Mych preferentially regulated genes involved in these stem cell-related biological processes and that their forced expression in the uninjured retina is sufficient to drive MG proliferation.

Although Mycb and Mych preferentially regulated many of the same top biological processes associated with reprogrammed and proliferating MG in the injured retina, biases were noted with Mycb preferentially regulating genes related to DNA synthesis and cell division, and Mych preferentially regulating genes related to ribosome biogenesis and protein synthesis. It is interesting that Mycb preferentially regulated genes associated with MG cell division, yet MG proliferation is more highly suppressed in injured *mych^mut^* retinas. This is likely due to the higher intrinsic apoptotic activity of Mych, which increases neuron death in the injured retina. This increased neuron death will stimulate more MG to reprogram and stimulate accumulation of microglia at sites of retinal injury, which will stimulate MG proliferation. In addition, because translation precedes and regulates cell division (Øvrebø et al., 2022), and because Mych regulates more translation-related genes than Mycb, Mych-target genes are more likely to couple translation with cell division.

Finally, it is worth noting that Mych is a result of a gene duplication event and is found only in ray-finned fishes. Mych confers a pro-regenerative advantage to the zebrafish retina beyond that of other Myc family members, which are conserved with mammals. Thus, through whole-genome duplication, some duplicated genes may gain pro-regenerative functions and further identification and characterization of these genes is warranted to understand their contribution to retina regeneration.

In conclusion, our study indicates that Mycb and Mych stimulate the expression of MG genes that control most of the top cellular processes enriched in MG as they acquire a retinal stem cell state. Consistent with regulating these stem cell-associated gene expression programs, forced expression of *mycb* or *mych* in the uninjured retina stimulates MG proliferation. Thus, when thinking about recapitulating the zebrafish's regenerative response in mammals, manipulations that target these genes and/or biological processes should be considered.

## MATERIALS AND METHODS

### Experimental models, genotyping and retinal injury

All animal work was approved by the University of Michigan's Institutional Animal Care and Use Committee. Zebrafish were housed in a recirculating water system maintained at ∼26°C. Room lights were on a 14/10 h light/dark cycle. This study used male and female fish at 4-12 months of age. Previously published transgenic fish lines used in this study were: *gfap:GFP* (Kassen et al., 2007), *1016 tuba1a:GFP* (Fausett and Goldman, 2006) and *ubi:Cas9* (Lee et al., 2020). New lines generated in this study were: *u6a:mycb-gRNA_1,2_*, *u6a:mych-gRNA_1,2_*, *hsp70l:mycb;cmlc:GFP*, *hsp70l:mych;cmlc:GFP*, *mycb^mut^* and *mych^mut^*.

Fin clips were used to isolate genomic DNA for genotyping fish. Briefly, fin clips were collected in 50 µl lysis buffer (10 mM Tris-HCl pH 8.0, 2 mM EDTA, 0.2% Triton X-100 and 100 µg/ml Proteinase K) and incubated at 55°C for 1 h with frequent vortexing and trituration. Samples were then heated to 95°C for 10 min and insoluble material removed by centrifugation at 1,500 ***g*** for 5 min. Next, 0.5 µl of genomic DNA was amplified by PCR using gene-specific primers (Table S1), and Radiant 2x RED Taq Master mix (Alkali Scientific, C223) in a 20 µl reaction volume. PCR products (5 µl) were analyzed on agarose gels with DNA size standards for determining amplified DNA size. For detecting gene edits, very small deletions, or fish harboring heterozygous mutations, a 10 µl PCR sample was used in T7E1 assay (±T7) as previously described (Vouillot et al., 2015).

Fish anesthesia was carried out by immersing fish in tricaine-containing fish water (75 mg/l) buffered to pH 7.2. Retinal injuries were either needle poke or intravitreal NMDA injection as previously described (Fausett and Goldman, 2006; Bernardos et al., 2007; Powell et al., 2016; Sahu et al., 2021). For all proliferation and immunofluorescence assays except those assaying dorsal or ventral proliferation, fish received two injuries/retina with one in a dorsal quadrant and the other a ventral quadrant of the retina. For analysis of dorsal and ventral MG proliferation, fish received one injury in either the dorsal or ventral retinal quadrant. For whole-retina RNA analysis or for RNAseq analysis of MG, fish received ten injuries/retina with five injuries/hemi-retina.

### Cell proliferation assays

For EdU labeling of proliferating cells, fish were injected IP with 10 µl of EdU (10 mg/ml) 3 h before sacrifice. Click-iT chemistry was used to detect EdU in retinal sections (Molecular Probes, 1511352). EdU^+^ cells were quantified in the INL where MG and proliferating MG-derived progenitors reside (Fausett and Goldman, 2006).

### RNA isolation, PCR and RNAseq

Fish were acclimatized to the dark overnight, anesthetized in tricaine, and then eyes were harvested and placed in a tray of ice-cold PBS for retinal dissection. Dissected retinas (generally three replicates) were placed into 500 µl of TRIzol (Invitrogen, 15596026) and dissociated by trituration using first a P200 PIPETMAN, and then a 1 ml syringe equipped with a 30 g needle. RNA was then purified using the manufacturer's (TRIzol, Invitrogen) recommendations. RNA concentration was determined on a NanoDrop One spectrophotometer (Thermo Fisher Scientific). In some instances, RNA was prepared from GFP^+^ MG that were purified by FACS from *gfap:GFP* transgenic fish retinas as previously described (Ramachandran et al., 2010a; Powell et al., 2013).

For cDNA synthesis, ∼1 µg of RNA was used with M-MLV or SuperScript III reverse transcriptase (Invitrogen, 18080085) according to the manufacturer's directions. cDNA was diluted 1/5 and 1 µl was used for PCR reactions as previously described (Fausett et al., 2008; Ramachandran et al., 2010a). We used a Luna qPCR Master Mix (NEB, M3003X) and an iCycler real-time PCR detection system (Bio-Rad) to carry out real-time PCR reactions. The ΔΔCt method was used to determine mRNA expression levels, and this was normalized to *gapdh_s_* for determining fold change in RNA expression*.*

For MG RNAseq studies, GFP^+^ MG were dissociated (Papain Dissociation system, Worthington Biochemical, LK003153) from 16 uninjured and 16 injured *gfap:GFP* fish retinas (ten needle poke injuries/retina) and GFP^+^ MG were purified by FACS as previously described (Ramachandran et al., 2010a; Powell et al., 2013; Allan et al., 2020). GFP^+^ MG were sorted directly into TRIzol LS (Invitrogen, 10296010) and total RNA was purified using Directzol RNA Microprep Kit (Zymo Research, R2060). RNA quality and quantity was analyzed on a 2200 TapeStation Bioanalyzer (Agilent). RNA RIN numbers were above 8 for samples used in this study. polyA RNA was used to prepare cDNA libraries that were generated by the University of Michigan's Advanced Genomics Core and DNA was sequenced on an Illumina NovaSeq 6000 platform. Sequencing reads were analyzed by the University of Michigan's Bioinformatics Core. Reads were mapped to the zebrafish genome (GRZc11). The number of reads for each expressed gene was determined and differentially expressed genes were restricted to those exhibiting at least a log2fold change >0.5-fold difference in expression with threshold abundance >5 fragments per kilobase of transcript per million mapped reads to eliminate very low abundant transcripts for which estimates of fold-change are unreliable. Differentially expressed genes had an adjusted *P*-value ≤0.05, FDR≤0.05.

For functional enrichment analysis, we used WebGestalt, a WEB-based gene set analysis toolkit (Liao et al., 2019).

### *In situ* hybridization assays

Digoxygenin-based *in situ* hybridization assays were performed as previously described (Barthel and Raymond, 2000). Fluorescence *in situ* hybridization assays used TSA PLUS CYANIN 3 kit (AKOYA Biosciences, NEL744001KT).

### CRISPR-based gene editing

We used CRISPRscan (https://www.crisprscan.org/) to identify gRNAs that target *mycb* or *mych* genomic DNA (Moreno-Mateos et al., 2015). gRNA DNA template synthesis using gRNA primer, universal primer, and *in vitro* transcription (Invitrogen, AM 1354) have been described (Vejnar et al., 2016). gRNA sequences are listed in Table S1. To identify the best gRNAs for gene editing, we injected single-cell zebrafish embryos with individual *in vitro*-transcribed gRNAs and Cas9-nanos-encoding mRNA (Invitrogen, AM1330). Two days later, some embryos were used to determine gene editing efficiency using T7 endonuclease 1 (T7E1) mismatch detection assays (Vouillot et al., 2015). gRNAs resulting in ∼50% or more gene editing were then used to make stable transgenic lines *u6a:mycb-gRNA_1,2_* and *u6a:mych-gRNA_1,2_* and then bred with *ubi:*Cas9*;cmlc:GFP* to generate double transgenic fish. Fish harboring indels were outbred to Wt fish for germline transmission and then inbred to drive mutations to homozygosity. See Table S1 for PCR primers and gRNAs.

### MO functional assays

Lissamine-tagged translation-blocking MOs were obtained from Gene Tools, LLC. For gene knockdown in adult fish, MOs were injected into the eye's vitreous fluid (0.5 µl) and electroporation used to facilitate cellular uptake as previously described (Fausett et al., 2008). See Table S1 for MO sequences.

### Heat shock and pharmacological inhibitors

In general, *hsp70l* promoters were activated by immersing fish in a 37°C water bath for 1 h, which was repeated every 6 h for the duration of the experiment. In some instances, a single 1 h heat shock was given. DAPT (Cayman Chemical, 13197) was used to inhibit Notch signaling and γ-secretase activity. DAPT was prepared in DMSO as a 10 mM stock and diluted 1/250 in fish water. Fish were exposed to drug or vehicle by immersion.

### Immunofluorescence, TUNEL and OPP assays

Twelve-micron-thick retinal sections were prepared using a Leica CM3050 S cryostat. Immunofluorescence was performed as previously described (Fausett and Goldman, 2006; Ramachandran et al., 2010a,b). Primary antibodies used in this study were: rabbit anti-GFP (Thermo Fisher Scientific, A6455; 1/1000); mouse anti-4C4 (1/500; gift from P. Hitchcock lab, University of Michigan, MI, USA; Becker and Becker, 2001); mouse anti-glutamine synthetase (GS) (Millipore Sigma, MAB302; 1/500); rabbit anti-HuC/D (Abcam, ab210554; 1/500); goat anti-PKC_β1_ (Santa Cruz Biotechnology, SC-209; 1/500); mouse anti-zpr1 (ZIRC, ZDB-ATB-081002-43; 1/500); rabbit anti-pS6 ribosomal protein (Ser235/236) (Cell Signaling Technology, 2211; 1/500). Secondary antibodies were: Alexa Fluor 555 donkey anti-mouse-IgG (H+L) (Thermo Fisher Scientific, A31570; 1/500); Alexa Fluor 555 donkey anti-rabbit IgG (H+L) (Thermo Fisher Scientific, A31572; 1/500); Alexa Fluor 488 donkey anti-mouse IgG (H+L) (Thermo Fisher Scientific, A21202; 1/500); Alexa Fluor 488 goat anti-rabbit IgG (H+L) (Thermo Fisher Scientific, A11008; 1/500); Alexa Fluor 568 donkey anti-goat IgG (H+L) (Thermo Fisher Scientific, A11057; 1/500).

An *In Situ* Cell Death Detection Kit, TMR red (Millipore Sigma, 12156792910) was used to detect TUNEL^+^ apoptotic cells.

For OPP labeling, the OPP Alexa Fluor™ 594 Protein Synthesis Assay Kit was used. One hour before sacrifice, fish received a 0.5 µl intravitreal injection of OPP (200 mM). Click-iT chemistry was used to detect OPP in retinal sections using the Click-iT Plus OPP Alexa Fluor 594 Protein Synthesis Assay Kit (Thermo Fisher Scientific, C10457).

### Microscopy and cell quantification

Images were captured using a Zeiss Axio Observer inverted epifluorescence microscope equipped with an Axiocam MRm camera, or a Leica DM2500 epifluorescence microscope equipped with a DFC7000T camera using Zeiss Zen 2.5 (Blue edition) and Leica LAS X software, respectively. Images were acquired using 20× dry objective. Single focal plane images were collected and imported into Adobe Photoshop for global adjustments of brightness and contrast, and manual quantification of cell numbers. Images were then imported into Adobe Illustrator for labeling and creation of multi-panel figures. EdU, 4C4 and TUNEL labeling was used to identify and quantify proliferating cells, microglia and apoptotic cells, respectively, in retinal sections as previously described (Fausett and Goldman, 2006; Ramachandran et al., 2010a; Wan et al., 2012, 2014; Wan and Goldman, 2017).

Retinal sections (12 µm) were prepared from 4% formaldehyde-fixed frozen tissue using a Leica CM3050 S cryostat. Sections from a single eye were serially distributed across four slides. To quantify EdU^+^, 4C4^+^ and TUNEL^+^ cells in injured retinas, we counted positive cells in every fourth section from a single needle poke injury site, or, in the case of NMDA damage, across the entire INL, and the sum of these values was multiplied by four to give the number reported on the *y*-axis in the graphs. For quantification of dorsal and ventral MG proliferation, we counted EdU^+^ and TUNEL^+^ cells in a 200 µm length of the INL that was 200 µm distance from the optic nerve head. In some cases, a representative image of a single section near the center of the injury site is shown to help visualize the injury response.

For lineage-tracing experiments, retinal sections from a single eye were distributed across four slides. Each slide was processed for immunofluorescence detection first for retinal cell type-specific markers and then click-iT chemistry to identify EdU^+^ cells. We quantified the total number of EdU^+^ cells and the number of co-labeled cell type marker^+^;EdU^+^ cells on each slide. Fluorescence intensity in retinal sections was quantified using ImageJ/Fiji.

### Statistical analysis

Unless otherwise indicated, sample size was four to eight retinas and experiments were repeated five or six times. Statistical analyses were performed in GraphPad Prism. The non-parametric two-tailed Mann–Whitney *U*-test was used for pairwise comparisons and Kruskal–Wallis test with Dunn's multiple comparison post-hoc test was used for multiple group comparisons. Statistical significance indicated in each graph as *P<0.05, **P<0.01, ***P<0.001, or ‘ns’ for not significant*.* All measurements were taken from distinct samples. Error bars represent s.d.

## Supplementary Material

10.1242/develop.203062_sup1 Supplementary information

Table S1. Primers, MOs, and gRNAs used in this study.

Table S2. Injury-induced DNA replication and cell cycle genes regulated by mycb and mych.

Table S3. Injury-induced ribosome biogenesis and protein synthesis genes regulated by mycb and mych.

Table S4. Injury and mycb regulated genes with insignificant regulation by mych.

Table S5. Injury and mych regulated genes with insignificant regulation by mycb.
