# Supplementary material for: Mycb and Mych stimulate Müller glial cell reprogramming and proliferation in the uninjured and injured zebrafish retina
Source: Development. 2024 Jul 26;151(14):dev203062. doi: 10.1242/dev.203062 (PMC11369687; doi:10.1242/dev.203062)
Supplement: Supplementary information [file develop-151-203062-s1.pdf]

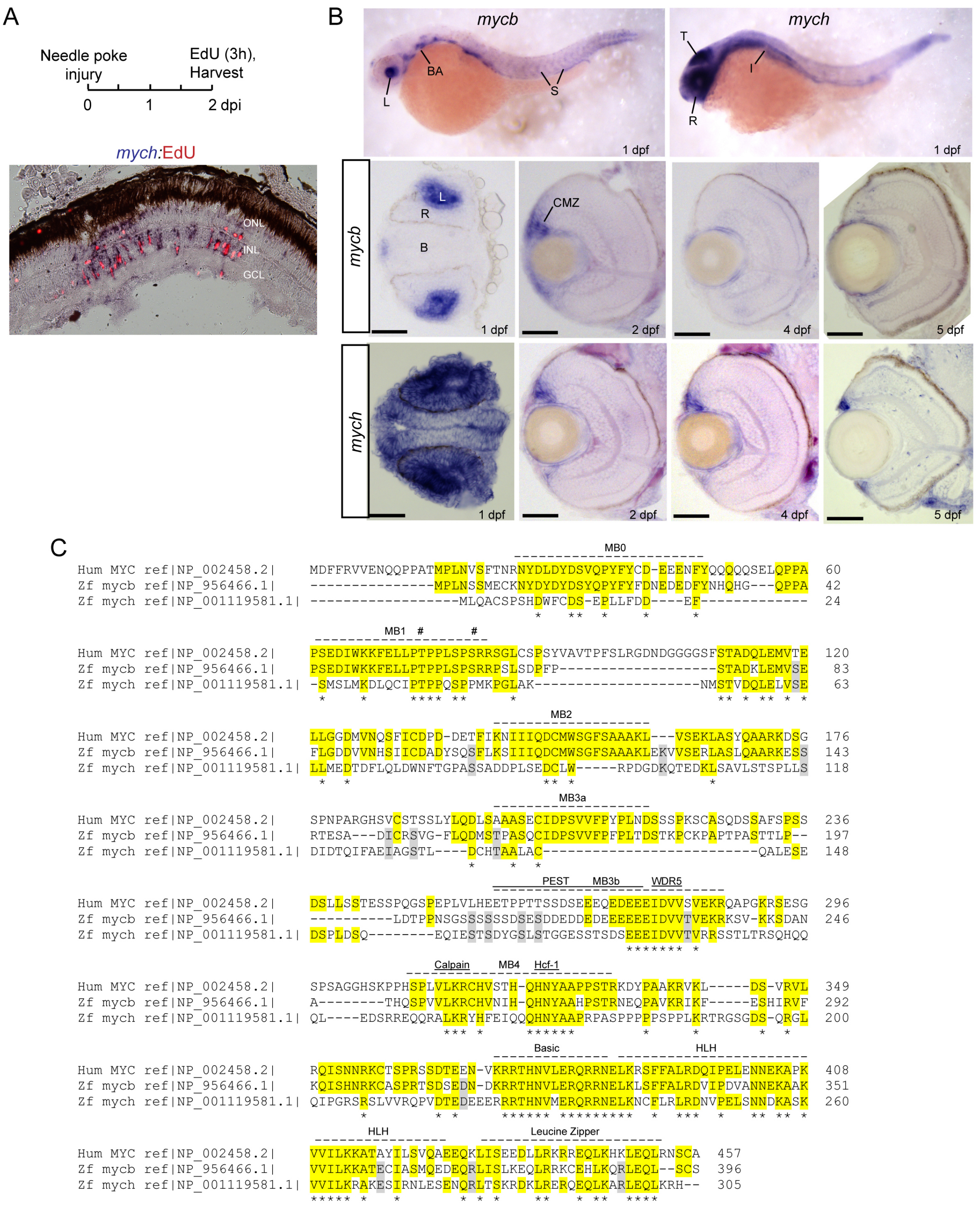

Fig. S1. Comparison of *mycb* and *mych* expression during zebrafish development and comparison of their amino acid sequences with human MYC. (A) Top is experimental timeline. *In situ* hybridization assay and EdU clicK iT chemistry reveal EdU+ (red) *mych* expressing (blue) cells in the INL of the needle poke injured retina at 2 dpi. (B) *mycb* and *mych in situ* hybridization assays on developing zebrafish. (C) Amino acids sequence conservation between human MYC and zebrafish Mycb and Mych. Abbreviations: ONL, outer nuclear layer; INL, inner nuclear layer; GCL, ganglion cell layer; dpi, days post injury; dpf, days post fertilization; L, lens; BA, branchial arch; R, retina; I, intestine; S, somite; T, tectum; B, brain; CMZ, ciliary marginal zone.

Development • Supplementary information

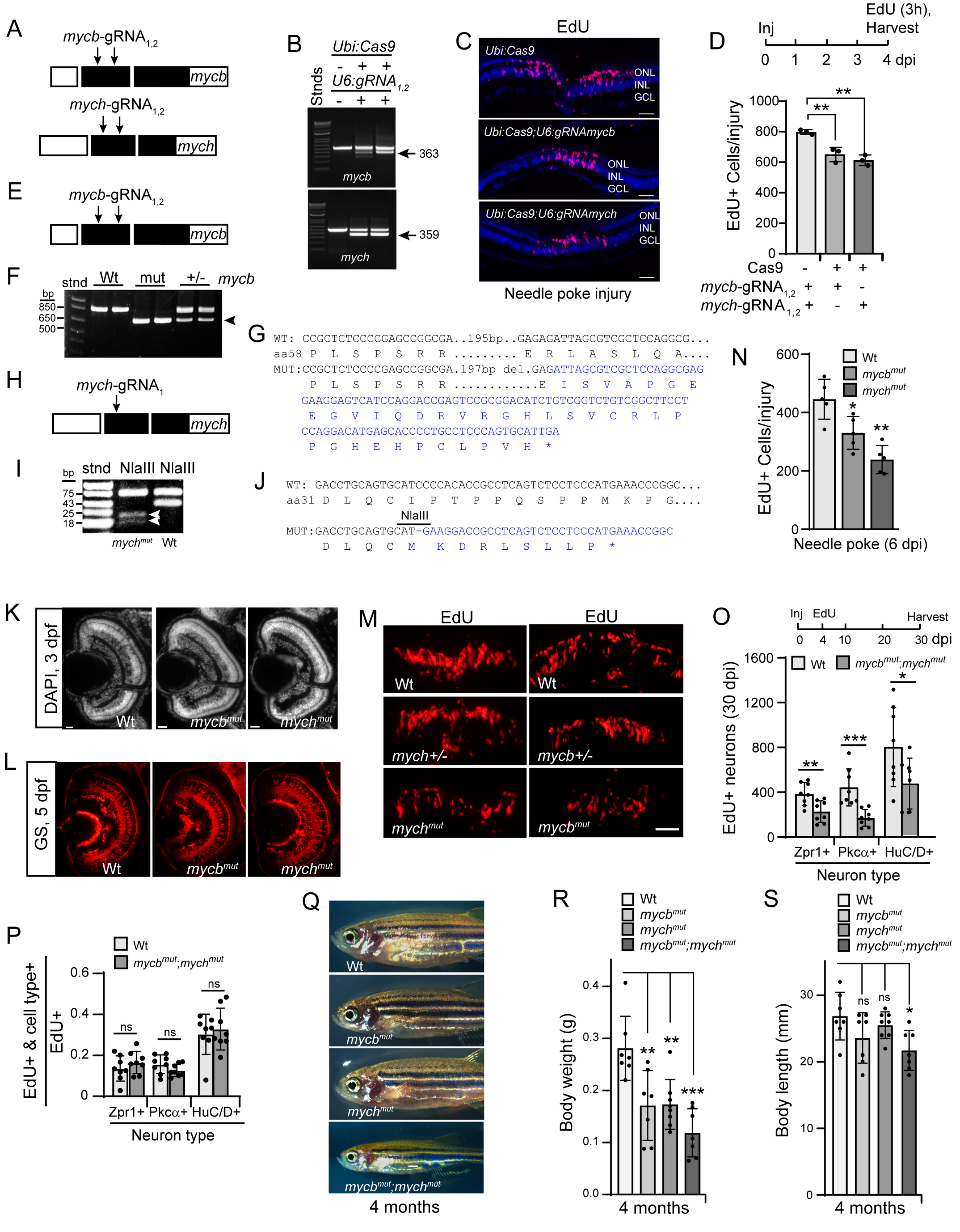

Development • Supplementary information

Fig. S2. *mycb^{mut}*, *mych^{mut}*, *hsp70l:mycb* and *hsp70l:mych* fish phenotypes. (A, E, H) Diagrams of *mycb* and *mych* gene exons and gRNAs used for gene editing. (B, F, I) Agarose gel showing expected gene edits (arrowhead). (C) EdU+ cells in needle poke injured F0 gene edited fish at 4 dpi. (D) Top is experimental timeline. Quantification of EdU+ cells in needle poke injured F0 gene edited fish at 4 dpi. (G, J) DNA sequencing identifies *mycb* and *mych* gene edits in germ-line transmissible *mycb^{mut}* and *mych^{mut}* fish. (K) DAPI staining reveals normal retinal lamination in Wt, *mycb^{mut}* and *mych^{mut}* fish at 3 dpf. (L) Glutamine synthetase (GS) immunofluorescence reveals normal MG differentiation in *mycb^{mut}* and *mych^{mut}* fish at 5 dpf. (M) Representative fluorescence images showing reduced EdU+ cells in *mycb^{mut}* and *mych^{mut}* fish compared to Wt fish following needle poke retina injury at 4 dpi. (N) Quantification of EdU+ cells/injury in needle poke injured fish at 6 dpi. (O) Top is experimental timeline. Graph is quantification of EdU+ neurons at 30 dpi (needle poke injury, EdU delivered at 4 dpi), reveals *mycb^{mut}*;*mych^{mut}* fish regenerate ~43% less EdU+ neurons than Wt fish. (P) Experimental timeline is as in (O), and sections were co-stained for EdU incorporation and retinal neuron specific markers (Zpr1 for photoreceptors; Pkca for bipolar neurons; and HuC/D for amacrine and ganglion cells). Note a similar percentage of EdU+ cells regenerate retinal neurons in Wt and mutant fish. (Q) Representative images of adult male Wt, *mycb^{mut}*, and *mych^{mut}* fish at 4 months of age. (R) Quantification of body weight in fish shown in (Q). (S) Quantification of body length in fish shown in (Q). (D) Mycb and Mych OE stimulate MG proliferation (arrows). Significant *P* values are **P*<0.5, ***P*<0.01, ****P*<0.001. Abbreviations: ONL, outer nuclear layer; INL, inner nuclear layer; GCL, ganglion cell layer; GS, glutamine synthetase; OE, overexpression; dpi, days post injury; dpf, days post fertilization; g, grams. Size marker is 100 microns (K, L), 2.8 mm (O, top 4 panels), and 3.5 mm (O, bottom 4 panels).

Development • Supplementary information

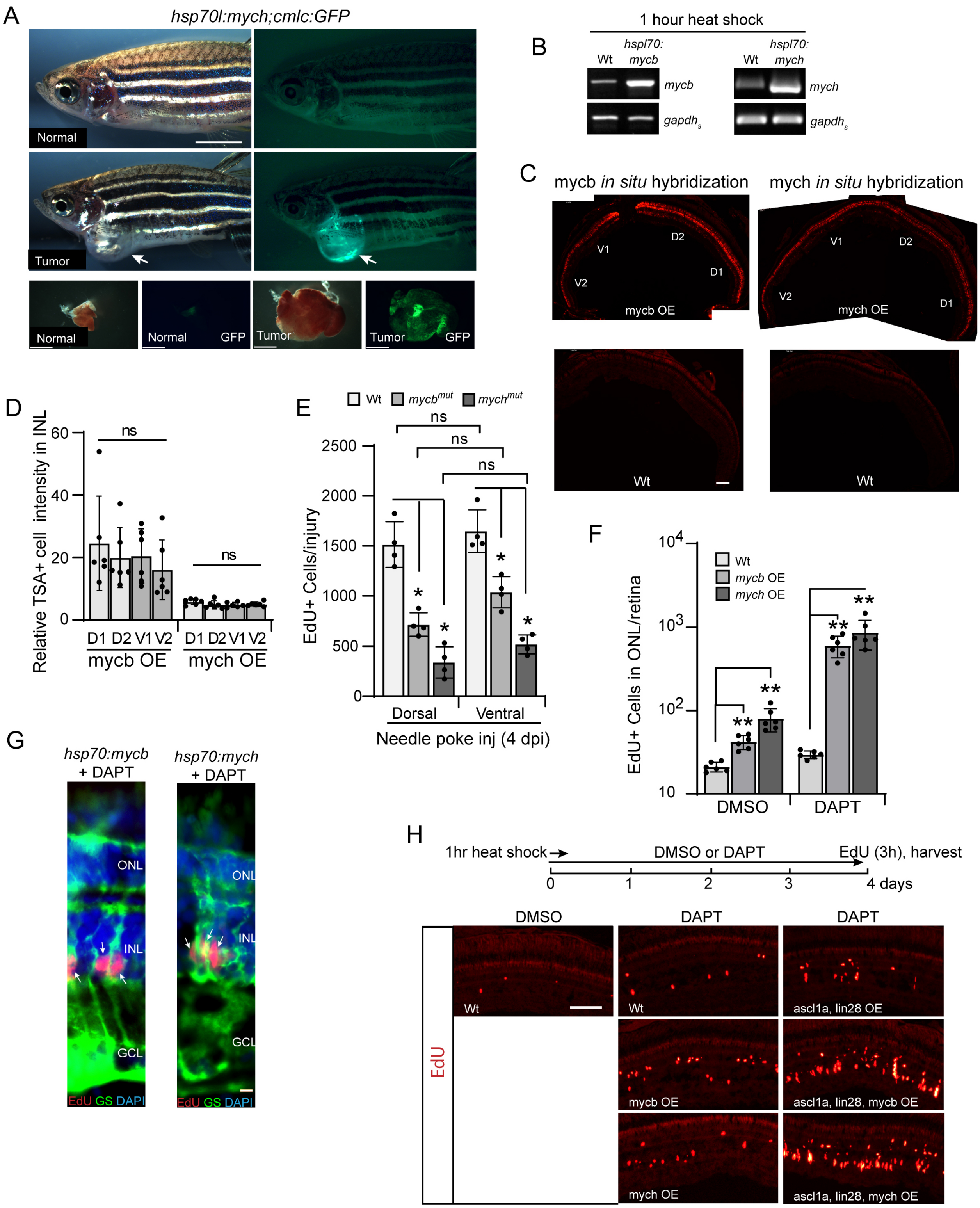

Development • Supplementary information

Fig. S3. Enhanced MG proliferation in Mycb and Mych overexpressing fish. (A) Comparison of Wt and tumor bearing *hsp70l:mych;cmlc:GFP fish*. Lower panels are dissected heart showing overgrowth and GFP expression. (B) PCR and agarose gel analysis shows a single 1h heat shock at 37 °C increases *mycb* and *mych* expression in *hsp70l:mycb* and *hsp70l:mych* fish, respectively. (C, D) Fluorescence *in situ* hybridization (C) and quantification of signal in INL (D) shows pan retinal expression without a dorsal or ventral bias, of *mycb* and *mych* from *hsp70l* promoters with a 1h heat shock. (E) Retinas were injured with a single needle poke to either the dorsal or ventral retina. At 4 dpi, EdU+ cells in the INL at each injury site were quantified. (F) Wt, *hsp70l:mycb,* and *hsp70l:mych* fish were either immersed in fish water containing DMSO or DAPT; fish received a 1h heat shock every 6h for 4 days and also an IP injection of EdU 3h prior to sacrifice. Graph shows quantification of EdU+ cells in ONL (putative rod progenitors). (G) Representative images of retinal sections from *hsp70l:mycb* and *hsp70l:mych* fish that received a 1h heat shock every 6h for 2 days before sacrifice and stained for EdU+ cells using clicK-iT chemistry. Retinal sections were stained for nuclei (DAPI, blue), glutamine synthetase (GS, green) and EdU (magenta). Arrows point to EdU+;GS+ MG in the INL. (H) Top is experimental timeline. Wt, *hsp70l:mycb, hsp70l:mych, hsp70l:ascl1a;hsp70l:lin28a, hsp70l:mycb; hsp70l:ascl1a;hsp70l:lin28a,* and *hsp70l:mych;hsp70l:ascl1a;hsp70l:lin28a* fish received a single 1h heat shock and then immersed in fish water containing DMSO or DAPT for 4 days. Three hours before sacrifice, fish received an IP injection of EdU. Retina sections were then collected and stained to detect EdU+ cells. Shown are representative images from each of the different fish genotypes showing Mycb and Mych synergize with DAPT-treatment and Ascl1a/Lin28a OE to stimulate MG proliferation in uninjured retina. Significant P values are *P<0.5, **P<0.01. The number of biological replicates is indicated by the dots in each graph. Abbreviations: Wt, wild type; ONL, outer nuclear layer; INL, inner nuclear layer; GCL, ganglion cell layer; GS, glutamine synthetase; IP, intraperitoneal; h, hour; ns, not significant. Size markers are 1 mm (A), 5 microns (D), and 100 microns (E, G).

Development • Supplementary information

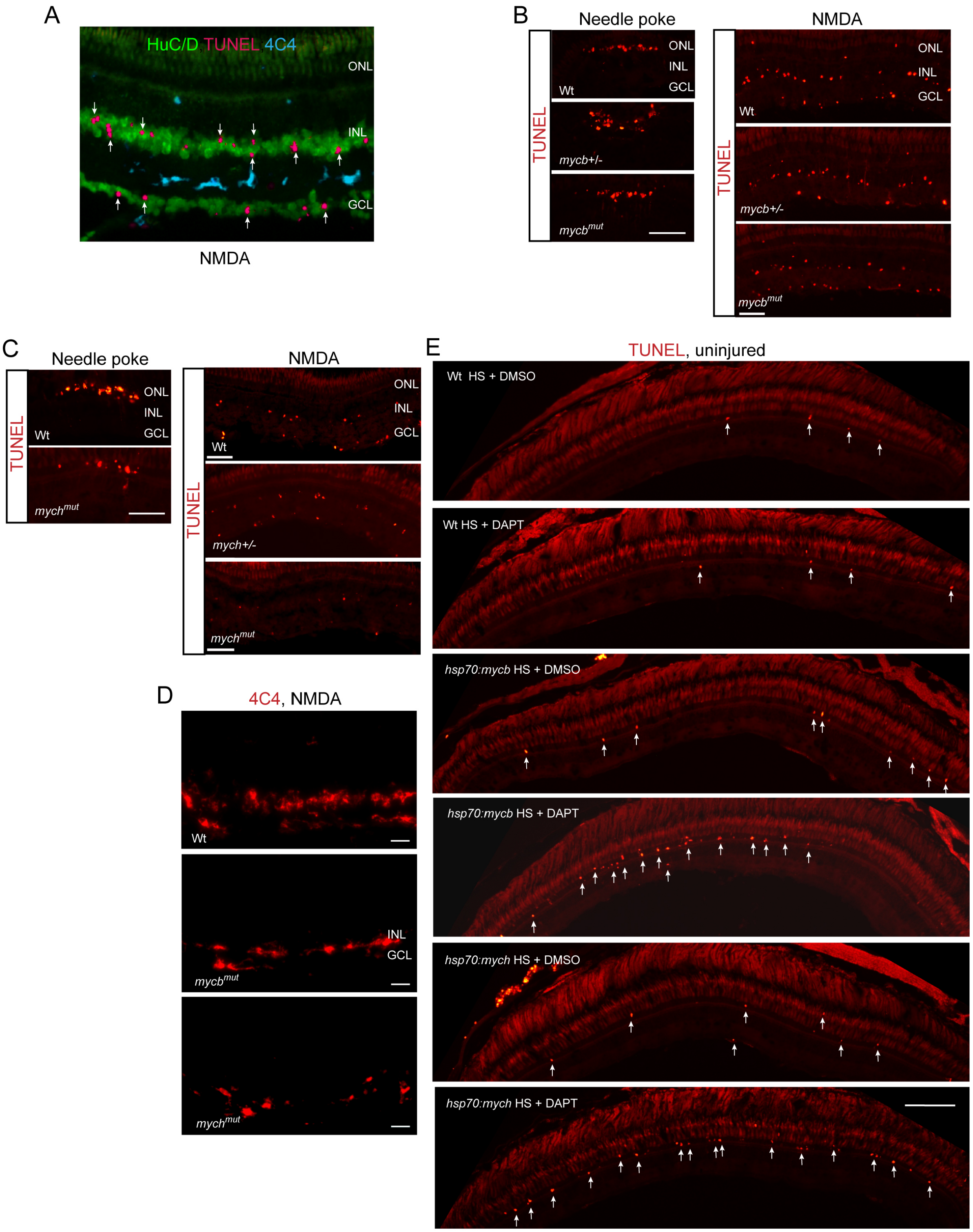
A
HuC/D TUNEL 4C4
ONL
INL
GCL
NMDA
B
Needle poke
NMDA
TUNEL
Wt
mycb+/-
mycb^mut
C
Needle poke
NMDA
mych^mut
mych+/-
D
4C4, NMDA
E
TUNEL, uninjured
Wt HS + DMSO
Wt HS + DAPT
hsp70:mycb HS + DMSO
hsp70:mycb HS + DAPT
hsp70:mych HS + DMSO
hsp70:mych HS + DAPT

Fig. S4. Endogenous Mych, and overexpressed Mycb and Mych stimulate neuron apoptosis in injured and uninjured retinas. (A) NMDA damaged retina stained with anti-HuC/D antibody to detect amacrine and ganglion cells (green fluorescence in INL and GCL); anti-4C4 antibody to detect microglia (blue ramified cells in inner plexiform layer); and TUNEL to detect dying cells (arrows point to dying cells; magenta). Note the only TUNEL+ cells are HuC/D+ neurons in the INL and GCL). (B-E) Representative fluorescence images stained for TUNEL (B, C, E) or 4C4 (D). (B, C) Endogenous Mych, but not Mycb, expression increases TUNEL+ cells in injured retina. (D) 4C4 immunofluorescence in Wt, $mycb^{mut}$, and $mych^{mut}$ fish. (E) TUNEL stain shows Mycb and Mych OE in uninjured retina synergizes with DAPT-treatment to increase neuronal apoptosis. Appreviations: Wt, wild type; ONL, outer nuclear layer; INL, inner nuclear layer; GCL, ganglion cell layer; HS, heat shock. Size markers are 100 microns (B, C, E), and 20 microns (D).

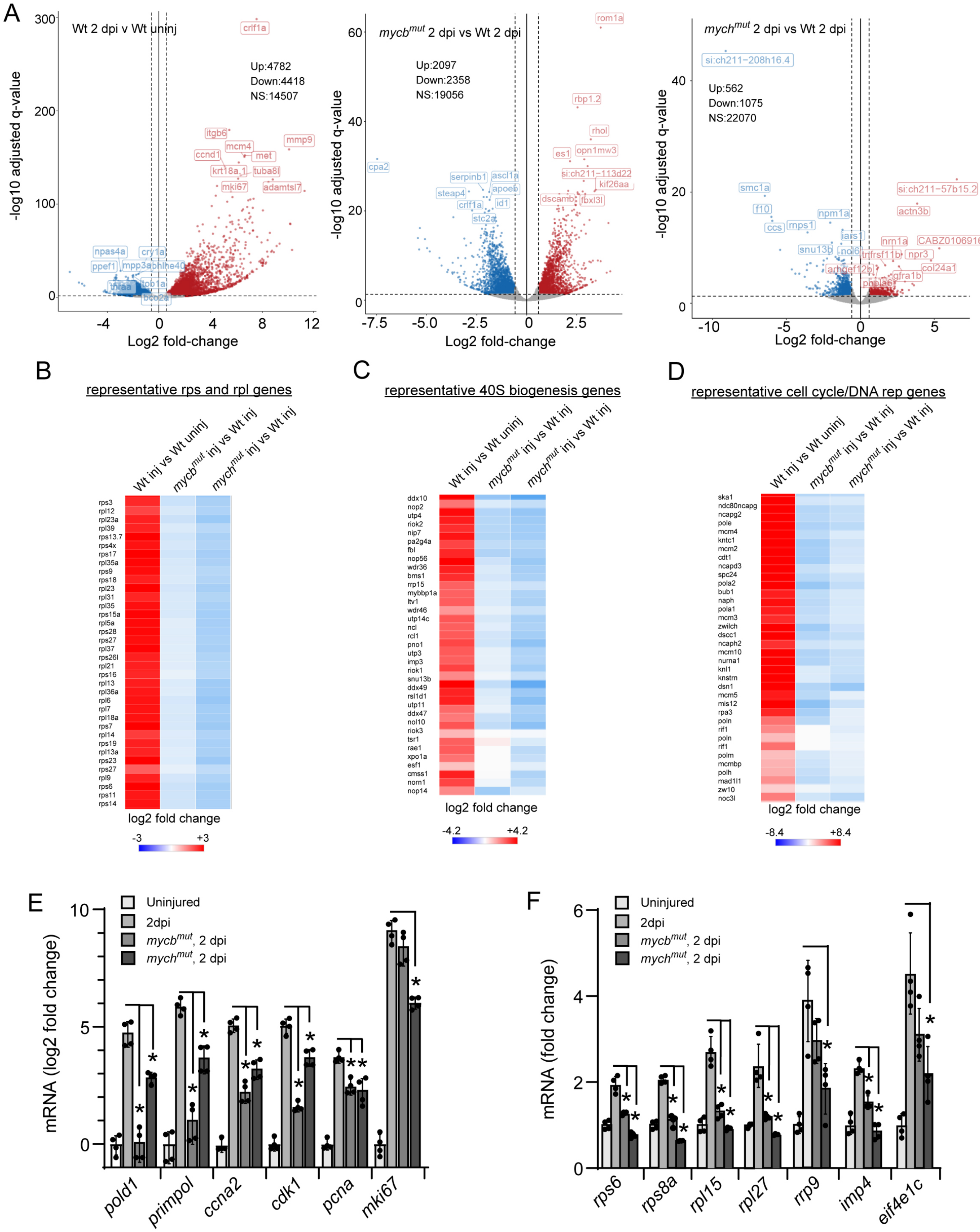

Development • Supplementary information

Fig. S5. Injury- and Mycb- or Mych-regulated genes in zebrafish MG. (A) Volcano plots of gene expression changes for indicate Wt and mutant fish lines. (B-D) Heat maps showing gene expression changes for representative ribosomal genes (B), 40s ribosome biogenesis genes (C), and cell cycle genes (D). (E, F) qPCR validation of select MG genes identified in MG RNAseq data sets that were induced with injury and regulated by Mycb and Mych. Significant P values are **P*<0.5. Although not indicated on the graphs, the difference in gene expression between uninjured and 2 dpi samples was statistically significant with *P*<0.5.

Table S1. Primers, MOs, and gRNAs used in this study.

Available for download at
https://journals.biologists.com/dev/article-lookup/doi/10.1242/dev.203062#supplementary-data

Table S2. Injury-induced DNA replication and cell cycle genes regulated by mycb and mych.

Available for download at
https://journals.biologists.com/dev/article-lookup/doi/10.1242/dev.203062#supplementary-data

Table S3. Injury-induced ribosome biogenesis and protein synthesis genes regulated by mycb and mych.

Available for download at
https://journals.biologists.com/dev/article-lookup/doi/10.1242/dev.203062#supplementary-data

Table S4. Injury and mycb regulated genes with insignificant regulation by mych.

Available for download at
https://journals.biologists.com/dev/article-lookup/doi/10.1242/dev.203062#supplementary-data

Table S5. Injury and mych regulated genes with insignificant regulation by mycb.

Available for download at
https://journals.biologists.com/dev/article-lookup/doi/10.1242/dev.203062#supplementary-data

Development • Supplementary information
